# Numerical Investigation on the Optimum Thermal Design of the Shape and Geometric Parameters of Microchannel Heat Exchangers with Cavities

**DOI:** 10.3390/mi11080721

**Published:** 2020-07-24

**Authors:** Haiwang Li, Yujia Li, Binghuan Huang, Tiantong Xu

**Affiliations:** National Key Laboratory of Science and Technology on Aero Engines Aero-Thermodynamics, Beihang University, Beijing 100191, China; 09620@buaa.edu.cn (H.L.); zy1804311@buaa.edu.cn (Y.L.); Huangbh@buaa.edu.cn (B.H.)

**Keywords:** microchannel heat exchanger, heat transfer enhancement, cavity, geometric parameters

## Abstract

Due to the large surface-area-to-volume ratio, microchannel heat exchangers have a higher heat transfer rate compared with traditional scale heat exchangers. In this study, the optimum microchannel cavity with high heat transfer and low flow resistance is designed to further improve microchannel exchangers’ thermal performance. A three-dimensional laminar flow model, consisting of Navier–Stokes equations and an energy conservation equation is solved and the conjugate heat transfer between the silicon basement and deionized water is taken into consideration. The impact of the shape, aspect ratio, size and spacing of the cavity on the thermal performance of microchannel exchangers are numerically investigated, respectively. The results indicated that the cavity on the sidewall can enhance heat transfer and reduce flow resistance simultaneously, and cavities with a relatively small expansion angle and streamlined edge could enhance thermal performance the most. Based on the conclusions, a new cavity shape is proposed, and the simulation results verify its excellent thermal performance as expected. Furthermore, investigation is performed to figure out the optimum design of the new cavity and the optimal geometric parameters of the cavity under different flow conditions have been obtained in principle for microchannel exchangers’ design.

## 1. Introduction

Due to the rapid augment in power density and miniaturization of electronic packages, there is a rising demand for effective cooling methods. As one of the most promising high efficiency heat exchange technologies, microchannel heat exchangers have a relatively larger surface-area-to-volume ratio and could provide a higher heat and mass transfer rate. Therefore, microchannel heat exchangers are used in the cooling of electronic devices, automotive heat exchangers and aerospace technology [1,2,3,4,5,6,7,8].

Turkerman and Pease [9] have investigated the flow and heat transfer characteristics in microchannel heat sink experimentally in the 1980s. They revealed that the microchannel heat sink has high efficiency to remove heat and the rate can reach up to 790 $W/{\mathrm{cm}}^{2}$. Steinke and Kandlikar [10] reviewed the available literature and generated a database to evaluate the availability of experiment data critically and they concluded that the Stokes theories for conventional fluid are applicable for microchannel flows.

The pioneering work of Turkerman and Steinke inspires a great deal of interest in the study of fluid flow and heat transfer in microchannel heat sinks.

Several numerical and experimental studies have been dedicated to investigate the flow characteristics and thermal performance in straight microchannels since the invention of microchannel heat sink [11,12,13]. In addition, scholars focus on the influence of the cross-sectional shape, Reynolds number, and hydraulic diameter of smooth microchannels [14,15,16,17]. The researchers found that although Nusselt number in microchannels has a slight augment compared with that of conventional channels, the simple straight microchannels can hardly improve the thermal performance since the relative higher pressure drop across the channel contributing to rising pumping power consumption.

Since the simple straight microchannel is unable to satisfy the rising demand of effective cooling, researchers have diverted their interest to passive techniques that have already been applied in conventional channels. Xie et al. [18,19] performed a numerical investigation to compare longitudinal and transverse wavy microchannels and they concluded that transverse wavy microchannels are much better than longitudinal microchannels in terms of pressure drop. Mohammed H.A et al. [20] and Dai et al. [21,22] revealed the superiority of zigzag microchannel in thermal performance with an expense of higher pressure drop in their studies and they also concluded that the periodic convergent-divergent construction can significantly enhance heat transfer. Ghaedamini et al. [23] investigated the effect of parameters in convergent-divergent microchannel numerically and concluded that increasing waviness can enhance heat transfer by producing chaotic advection by increasing the slow mixture.

As an efficient passive heat transfer enhancement method, the flow disruption techniques have also attracted the interest of scholarsb [24,25,26,27]. They carried out ribs and cavities to disrupt the working fluid, mix the fluid layers and improve the thermal performance eventually. The results showed that ribs could significantly improve the heat transfer performance with a higher pressure drop as a loss. Chai et al. [24] studied the effect of fan-shaped ribs on flow characteristics and heat transfer performance of microchannel heat sinks. The results indicated that ribs with relatively lower height and lager spacing have better thermal performance. The Nusselt number could increase by about 4–103% with an acceptable augment of friction factor. In their further study [25], they investigated the thermal performance and flow characteristics in microchannels with rectangular ribs. They obtained the optimal design of the rib dimensions and locations. Hong et al. [26] and Liu et al. [27] studied the microchannels with strip-fin and longitudinal vortex generators (LVGs). They revealed that these microchannel heat sinks could noticeably improve heat transfer accompanied. However, the pressure drop is much higher than smooth microchannel as well. In addition to microchannels with ribs, fluid flow and heat transfer in microchannels with cavities have also received considerable attention. Ahmad et al. [28] investigated the effect of geometrical parameters on flow characteristics and heat transfer in triangular, trapezoidal and rectangular microchannels numerically. They pointed out that trapezoidal grooves have shown the best thermal performance compared with other types of channels with increasing 51.59% of Nusselt number and 2.35% for friction factor. However, the augment of heat transfer is accompanied with the higher pressure drop. Xia et al. [29] analyzed the impact of geometric parameters on flow characteristics and heat transfer in the microchannel heat sink with fan-shaped and triangular cavities. They obtained the most optimal geometric parameters of the cavities in principle.

It is obvious from the literatures review that many studies have been done to investigate the effect of cavities on microchannel heat sink thermal performance. In addition, there is no doubt that interrupting the boundary layer and inducing the mainstream separation to form vortices are two significant means to enhance heat transfer performance in microchannel heat sinks. However, the following issues still need to be figured out: (1) Most of the investigation of the above literatures were under the flow condition for Re≥200. However, due to the small hydraulic diameter of the microchannels and the slow flow rate, the working Reynolds number may be relatively lower in some cases. Hence, there is a lack of conclusions for relatively lower Reynolds numbers. (2) The existing literatures mostly focused on the study of cavities of a certain shape, lacking the lateral comparison of different shapes and the influence mechanism of cavity shape. (3) A new design is needed that provides higher heat transfer ability with a relatively low pressure drop. Therefore, this paper numerically explores the impact of the cavity shape on the thermal performance in microchannels. Then, based on the conclusion, we propose a new shape of the silicon-based microchannel array with periodic cavities and carry out a simulation to investigate the optimized design for the geometric parameters. 

## 2. Computational Method

### 2.1. Computational Domain and Boundary Conditions

**Figure 1:**
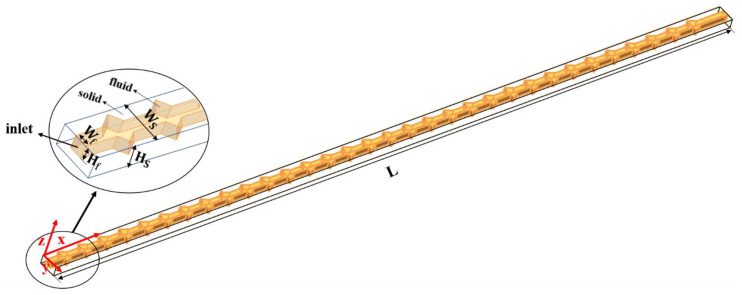
Structure of microchannel heat sink.

**Figure 2:**
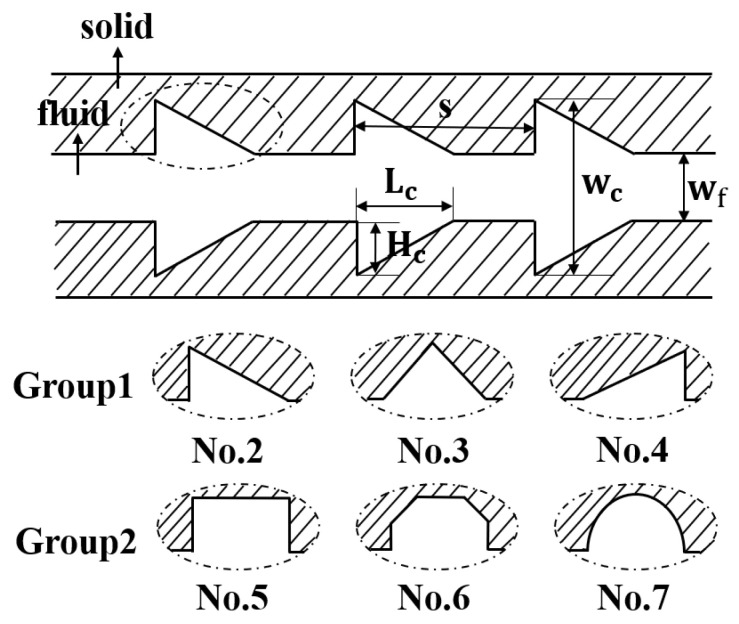
Structures of cavities.

Figure 1 illustrates the computational domain, corresponding key notions as well as the coordinate system used in the present work. The length (*L*), width ($W_{s}$) and height ($H_{s}$) of the silicon basement are 40, 1, and 0.5 mm, respectively. The width ($W_{f}$) and height ($H_{f}$) of the microchannel are both 0.3 mm. Figure 2 shows the detailed cavities’ structures of the cross-section in this study. In order to investigate the effect of cavity shape on thermal performance of microchannel heat sink, two groups of silicon microchannels are investigated. Group 1 is intended to explore the impact of expansion angle of the cavity, including forward triangular (No. 2), isosceles triangular (No. 3) and backward triangular (No. 4); while Group 2 consists of rectangular (No. 5), rectangular-trapezoid (No. 6) and semicircular (No. 7) to figure out the influence of the degree of streamlined edges. It should be noted that No. 1 is simple straight microchannel that severs as a comparison to figure out how the existence of cavity affect the flow structure and heat transfer. All the depths of the cavities are 0.2 mm in *y*-direction, and the lengths in the *x*-direction are 0.4 mm. The distance between two adjacent cavities is 0.8 mm in the flow direction (*x*-direction).

The working fluid is deionized water, and silicon is severed as a solid material. The Reynolds number ranges from 20 to 500. The boundary conditions are set as follows:

For the inlet, velocity is assumed to be uniform:

(1) $$x=0,\;u=u_{in},\;T_{f}=T_{in}=298.15K$$

where $u_{in}$ and $T_{in}$ are given inlet velocity and temperature at the microchannel inlet. The pressure-outlet boundary condition is applied at the microchannel outlet:

(2) $$x=L,\;p_{f}=p_{out}=1\;atm$$

where $p_{out}$ is the pressure of the outlet.

For the contact surface of inner wall and fluid,

(3) $$u=v=w=0,-k_{s}\frac{\partial T_{s}}{\partial n}=-k_{f}\frac{\partial T_{f}}{\partial n}$$

where *n* is the local coordinate normal to the wall.

For the base of silicon basement,

(4) $$z=-0.5,-k_{s}\frac{\partial T_{s}}{\partial n}=q_{w}$$

where $q_{w}$ is the heat flux.

For other surfaces,

(5) $$\frac{\partial T_{f}}{\partial x}=0\;or\;\frac{\partial T_{s}}{\partial x}=0$$

### 2.2. Mathematical Foundation

The value of Knudsen number ($K_{n}$) is an important parameter to measure whether the flow satisfies the continuity assumption, which is defined as:

$$K_{n}=\frac{\lambda }{l}$$

where λ refers to the molecular free path of the working fluid and *l* represents the characteristic length of the structure. In this paper, deionized water was applied as working fluid. For fluid and solid, the values of λ are considered to be equal with their molecular diameters due to the compact arrangement of the molecular. Therefore, in this study:

$$K_{n}=\frac{\lambda }{l}=\frac{\lambda }{D_{h}}=\frac{4\times 10^{-10}\;m}{3\times 10^{-6}\;m}=1.33\times 10^{-4}<10^{-3}$$

When $K_{n}$<$10^{-3}$, the flow is considered to satisfy the continuity assumption. Therefore, the continuity assumption is applied in this paper. 

In addition, the numerical model for fluid flow within the microchannel is processed under the following assumptions:

(1) Steady, incompressible, laminar, three-dimensional fluid flow.
(2) The effect of body forces is negligible.
(3) The radiation heat transfer is neglected.

Based on the above assumptions, the governing equations can be written as given below in tensor form: 

Continuity equation:

(6) $$\frac{\partial }{\partial x_{i}}\left(\rho_{f}u_{i}\right)=0$$

Momentum equation:

(7) $$\frac{\partial }{\partial x_{i}}\left(\rho_{f}u_{i}u_{j}\right)=-\frac{\partial p}{\partial x_{j}}+\frac{\partial }{\partial x_{i}}\left[\mu_{f}\left(\frac{\partial u_{j}}{\partial x_{i}}+\frac{\partial u_{i}}{\partial x_{j}}\right)\right]$$

Energy equation:

(8) $$\frac{\partial }{\partial x_{i}}\left(\rho_{f}u_{i}c_{pf}T\right)=\frac{\partial }{\partial x_{i}}\left(k_{f}\frac{\partial T}{\partial x_{i}}\right)+\mu_{f}\left[2{\left(\frac{\partial u_{i}}{\partial x_{i}}\right)}^{2}+{\left(\frac{\partial u_{j}}{\partial x_{i}}+\frac{\partial u_{i}}{\partial x_{j}}\right)}^{2}\right]$$

For the substrate conduction:

(9) $$\frac{\partial }{\partial x_{i}}\left(k_{s}\frac{\partial T}{\partial x_{i}}\right)=0$$

where ρ is density, μ is dynamic viscosity, $c_{p}$ is specific heat capacity, k is thermal conductivity, and *x*, *y*, *z* are *x*, *y*, *z* coordinates. Subscripts *s* and *f* refer to solid and fluid, respectively.

There are four parameters of interest in the present work, including the Reynolds number (*Re*), friction factor (*f*), Nusselt number (*Nu*) and thermal enhancement factor (*ƞ*). 

The Reynolds number is defined as:

(10) $$Re=\frac{\rho_{f}u_{m}D_{h}}{\mu_{f}}$$

where $u_{m}$ is the average velocity, $D_{h}$ is the hydraulic diameter of the microchannel and $\mu_{f}$ represents the viscosity of the working fluid. It should be noted that the value of hydraulic diameter in the constant cross-section segment, which is defined as $D_{h}=2WH/\left(W+H\right)$, is adopted for all microchannels in this study. 

The average fiction factor (*f*) is calculated by the pressure drop (∆p) across the flow direction of the microchannel as:

(11) $$f=\frac{2∆pD_{h}}{\rho_{f}Lu_{m}{}^{2}}$$

where *L* is the whole length of the microchannel.

The local Nusselt number (*Nu*) is defined as:

(12) $$Nu\left(x\right)=\frac{h\left(x\right)D_{h}}{k_{f}}$$

where *h(x)* represents the heat transfer coefficient that is defined as:

(13) $$h\left(x\right)=\frac{q_{w}W_{s}L}{A\left[T_{w}\left(x\right)-T_{f}\left(x\right)\right]}$$

where $q_{w}$ is the heat flux at the base surface of the silicon basement, A is the area of contact surface between the fluid zone and solid zone. $T_{w}\left(x\right)$ and $T_{f}\left(x\right)$ are the local conduction wall temperature and local qualitative temperature of working fluid, respectively. $T_{f}\left(x\right)$ is defined as:

(14) $$T_{f}\left(x\right)=\frac{1}{2}\left[T_{in}\left(x\right)+T_{out}\left(x\right)\right]$$

The thermal enhancement factor (*ƞ*) is adopted to access the enhanced heat transfer surfaces in heat exchange design. According to Webb [30] and Karwa [31], the performance enhancement factor is defined as the ratio of the heat transfer coefficient of the microchannel with cavities to the simple straight microchannel under equal pumping power

(15) $$ƞ=\frac{h}{h_{1}}=\frac{Nu/Nu_{1}}{{\left(f/f_{1}\right)}^{1/3}}$$

### 2.3. Solutions Methods and Convergence Criteria

The governing equations are solved using commercial code FLUENT 19.0. A second-order upwind differencing scheme is applied to discretize the code and SIMPLE algorithm, and the finite-volume approach is used to solve the equations. The solutions are considered to be converged when the normal residual values were less than $10^{-5}$ and $10^{-7}$ for energy equation. 

A grid independence procedure for No. 2 is implied before the simulation carry out. The Reynolds number (*Re*) is 200 while the cell number is varied from 1.57 million to 4.78 million. The relative error is calculated as the following equation:

(16) $$e\%=\left|\frac{J_{2}-J_{1}}{J_{1}}\right|\times 100$$

where *J* represents any parameters including temperature, Nusselt number and pressure drop, $J_{1}$ represents the value of the parameter calculated from the finest grids, while $J_{2}$ represents that acquired from other grids. Table 1 shows the relative errors of Nusselt number (*Nu*) and pressure drop (∆p) of different grids compared with the finest grids of 4.78 million cells. It is obvious from this that the grid numbers of 2.97 million has a reasonable accuracy and has been used in the current study.

## 3. Results and Discussion

### 3.1. Validation for the Smooth Microchannel Heat Sink

In order to verify the accuracy and reliability of numerical methods, verifications of the smooth microchannel are performed. According to Steinke and Kandlikar [10], the laminar flow friction factor of rectangular microchannels could be calculated as follows:

(17) $$f=f_{FD}+\frac{KD_{h}}{L}$$

(18) $$f_{FD}Re=96\left(1-1.3553AR+1.9467AR^{2}-1.7012AR^{3}+0.9564AR^{4}-0.2537AR^{5}\right)$$

(19) $$K=0.6797+1.2197AR+3.3089AR^{2}-9.5921AR^{3}+8.9089AR^{4}-2.9959AR^{5}$$

where *AR* is the aspect ratio of the microchannels.

The temperature difference between the inlet and outlet is obtained from energy balance:

(20) $$T_{out}-T_{in}=\frac{q_{w}W_{s}L}{\rho A_{in}u_{m}C_{p}}$$

where $A_{in}$ and $C_{p}$ are the inlet area of the microchannel and the specific heat capacity of deionized water, respectively.

**Figure 3:**
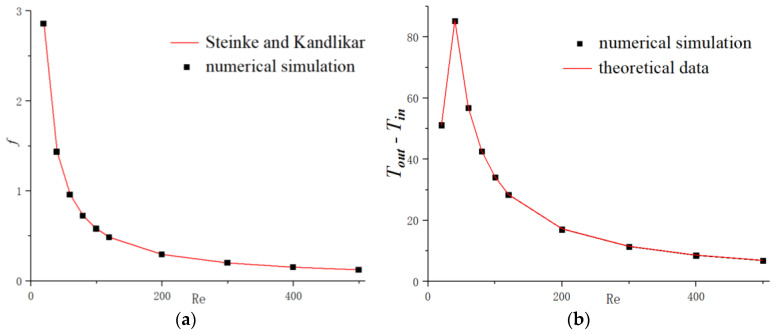
Comparison of numerical results with theoretical data for smooth microchannel heat sink. (**a**) *f*; (**b**) $T_{out}-T_{in}$.

The results in the present work compared with the theoretical data obtained from Equations (17) and (20) are shown in Figure 3a,b, respectively. It can be clearly seen that the numerical results keep excellent agreement with the theoretical data and the maximum relative error is less than 1.7% for friction factor, and 1.3% for temperature discrepancy between inlet and outlet. It should be mentioned that for Re=20, the heat flux is 30,000 $W/m^{2}$ and 100,000 $W/m^{2}$ are applied for other flow conditions and this discrepancy has no effect on the interested parameters that we focus on. Therefore, the present numerical code is able to simulate the fluid flow and heat transfer performance for the microchannel heat sinks.

**Figure 4:**
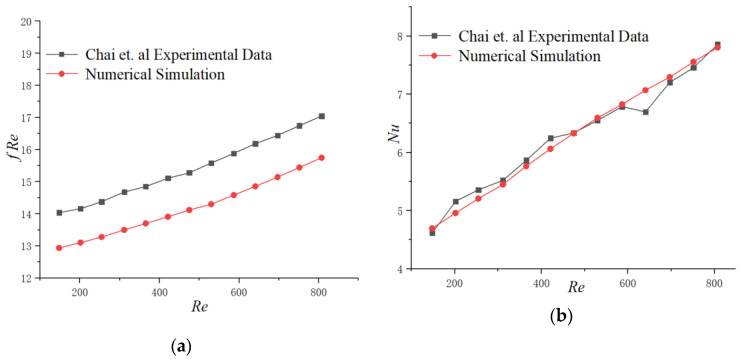
Comparison of numerical results with experimental data [25]. (**a**) *fRe* (**b**) *Nu*.

In addition, we conducted a numerical simulation of the tested microchannel in [32] to verify the simulation methods by comparing the simulation results with the experimental data in [32]. The test section is composed with a silicon basement and microchannels. The width of the microchannel is 0.1 mm, the depth is 0.2 mm and the whole thickness is 0.35 mm. The Reynolds number ranges from 147.68 to 807.14. Figure 4 presents the simulation results compared with the experimental data in [32] and the relative error of fRe and *Nu* are within 8.95% and 5.57%, respectively.

### 3.2. The Effect of Cavity Shape

#### 3.2.1. Flow Characteristics

**Figure 5:**
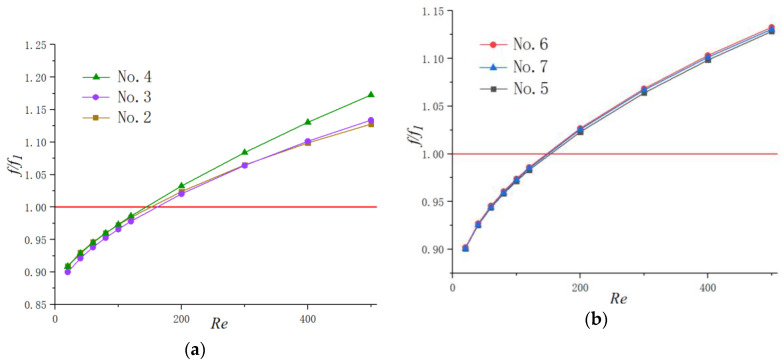
Variation of $f/f_{1}\;$ with Re. (**a**) Group 1, (**b**) Group 2.

Figure 5 illustrates the effect of Reynolds number on the average friction factor with respect to the straight baseline microchannel (No. 1). It can be seen that the values of $f/f_{1}$ increase with the augment of the Reynolds number and the difference among them turns to become more obvious with the increase of Reynolds numbers. Additionally, for lower Reynolds numbers (Re≤120 in present work), the value of $f/f_{1}$ is under 1, indicating that the heat sink with periodic cavities can reduce the flow friction in some level. This phenomenon may be contributed to the following reasons. Unlike flow in straight microchannel, when the fluid flows into the microchannel with cavities, the viscous resistances exist not only between the fluid and microchannel walls but also between the fluid and fluid since the main stream slips over the cavities. The friction between fluid and fluid is much lower than that between fluid and static walls. Additionally, the jetting and throttling effects are not obvious for lower Reynolds number. Therefore, the friction of microchannels with cavities is lower than that of simple straight microchannels in the above situation. However, with the increase in mainstream velocity, the viscous dissipation causing by spurt and throttling effects turns to be more significant. Hence, the fiction factors in microchannels with cavities surpass that of simple straight microchannels.

It can also be seen from Figure 5a that the friction factor of the No. 4 channel is much higher than that of the other two channels in Group 1. Additionally, the differences of relative friction factors ($f/f_{1}$) among the microchannels with different shape cavities are negligible for relatively lower Reynolds numbers. However, the disparity tends to be more obvious with the augment of the Reynolds number. For example, when *Re* = 40, the relative difference between No. 2 and No. 4 is 0.125%; when *Re* = 400, the discrepancy gets up to 2.89%. As for friction factors in Group 2, the discrepancy is not obvious, with only 0.383% for *Re* = 500. 

**Figure 6:**
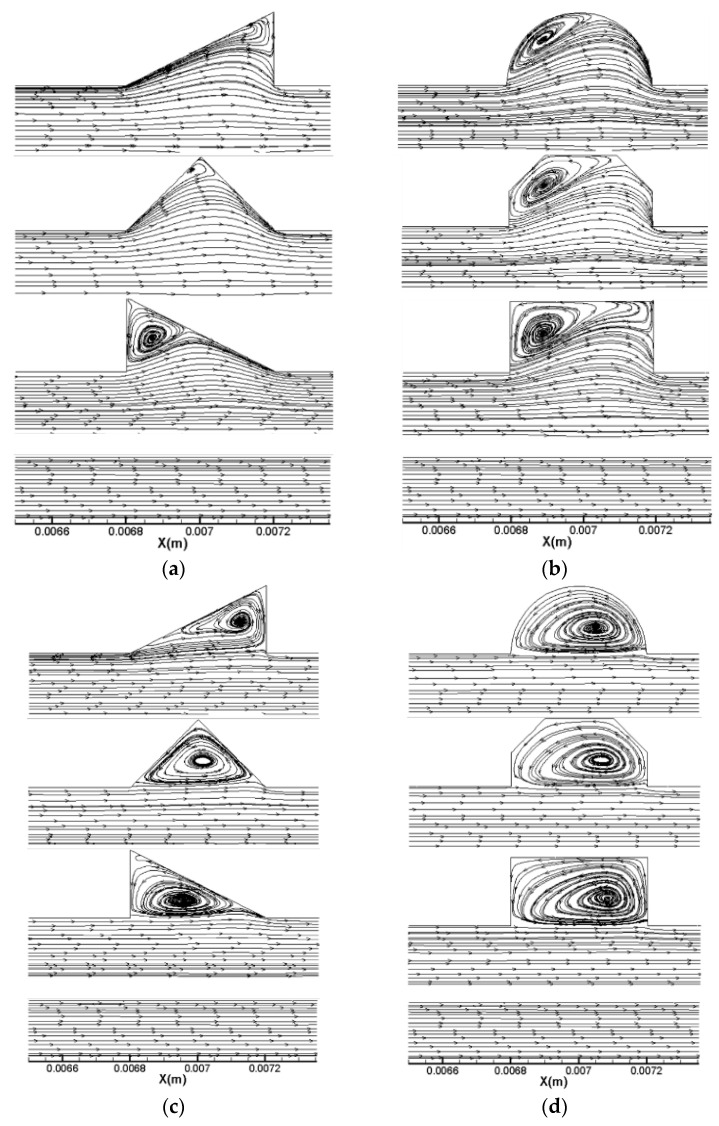
Streamlines in a heat sink for *Re* = 20 and *Re* = 400. (**a**) *Re* = 20 Group 1, (**b**) *Re* = 20 Group 2, (**c**) *Re* = 400 Group1, (**d**) *Re* = 400 Group 2.

**Figure 7:**
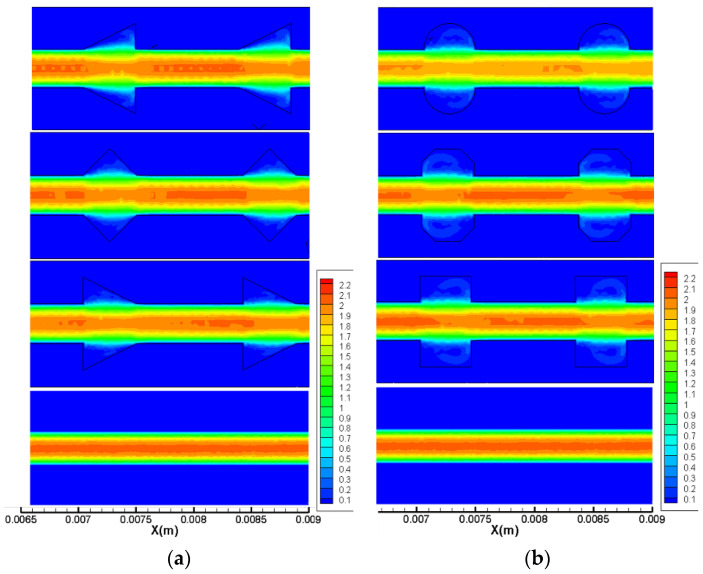
Local velocity distribution for *Re* = 400. (**a**) Group 1, (**b**) Group 2.

Flow fields analysis is carried out to explain the above phenomenon. The streamlines at *Re* = 20 and *Re* = 400 along the plane of half the working fluid depth (*z* = −0.15 mm) are displayed in Figure 6 for all microchannels. As observed in the figure, the following phenomena could be identified. (1) When *Re* = 20, for the cavities with a relatively larger expansion angle, the effect of the reverse pressure gradient is more significant due to the sudden expansion; hence, the recirculation area inside No. 2 is much larger than that in No. 3 and No. 4. (2) When the Reynolds number becomes as large as 400, all microchannels show jetting and throttling effects, and vortex structures could be observed in cavities. The local velocity distribution for *Re* = 400 for two groups are shown in Figure 7. Since the velocity gradient in *y* direction can suggest the mixing level between the hot water near the wall and the cold water in the channel center, the smaller the velocity difference in *y* direction, the better the mixture of the fluid. As can be observed from Figure 6, the longitudinal velocity gradient is lower in No. 7, indicating that the fluid layers are strongly mixed in microchannels with streamlined-edged cavities, which may improve the thermal performance as well. 

#### 3.2.2. Heat Transfer

**Figure 8:**
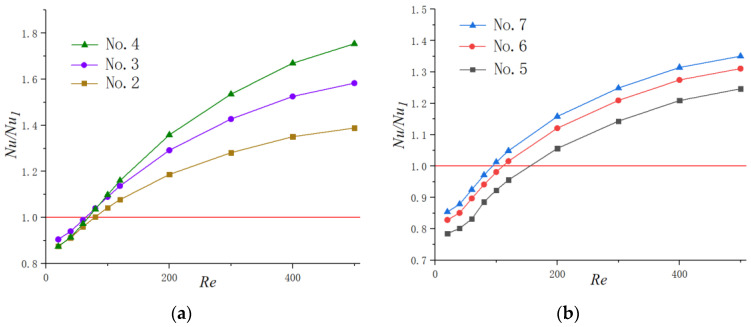
Variation of $Nu/Nu_{1}\;$ with Re. (**a**) Group 1, (**b**) Group 2.

Figure 8 presents the effects of the Reynolds number on $Nu/Nu_{1}$ for microchannels with a different shape of cavities. It is worth noting that the values of $Nu/Nu_{1}$ are under 1 for the lower Reynolds number in all heat sinks with periodic cavities. This may be contributed to by the following reasons: when the flow rate is constant, due to the local expansion of the microchannel, the addition of cavities will cause a decrease in the average velocity of the fluid; hence, the convective heat exchange effect between the cold fluid and hot wall may be weakened. In addition, for working conditions with a relatively small velocity, the fluid slips over cavities instead of generating jetting and spurting effects inside them. Therefore, for *Re* < 100, cavities have no positive effect on the heat transfer enhancement of microchannel heat sinks, and straight microchannels are optimum for microchannel thermal design. However, the tendency increased significantly with the augment of the Reynolds number and exceeds the value of 1, which severed as a red line in the figures, eventually. This suggests that the present microchannels with cavities can improve the heat transfer performance only for higher Reynolds number conditions, and the enhancement degree increases with the augment of the Reynolds number. For instance, when *Re* = 100, the *Nu* in No. 4 is 1.10 times that of simple straight microchannels (No. 1), and this value turns to become 1.75 for *Re* = 500. As for the situation of relative lower flow rate, there is few enhancements. This could be contributed to the fact that there are less jetting and throttling effects when the flow rate is small. Additionally, the fluid velocity is too slow, meaning that the fluid slips into the cavities rather than inducing the secondary flows in them like higher flow velocity does. It is also interesting to note that the value of *Nu* in No. 7 is higher than that of the other two microchannels in Group 2 for a certain Reynolds number, though the heat transfer area for No. 7 is smaller that of No. 5 and No. 6. This indicates that enlarging the heat transfer area is not the most effective way to enhance heat transfer in some situations. It should be mentioned that the values of the average Nu number obtained in this study for some conditions are lower than that of traditional scale channels. This phenomenon was also observed in other studies of microchannel thermal performance [33,34] and we thought it may be contributed to by the following fact. The *Nu* number could be considered as the ratio of conduction thermal resistance $D_{h}/\lambda_{f}$ to convection thermal resistance 1/h. Compared with channels on a conventional scale, the hydraulic diameter ($D_{h}$) of microchannels is much smaller; hence, the temperature gradient in the direction normal to the wall is much steeper and the thermal conduction is more drastic. Therefore, the magnitudes of conduction thermal resistance are lower than that of traditional scale channels, indicating that the thermal conductivity in the fluid is important for heat transfer in microchannels. 

**Figure 9:**
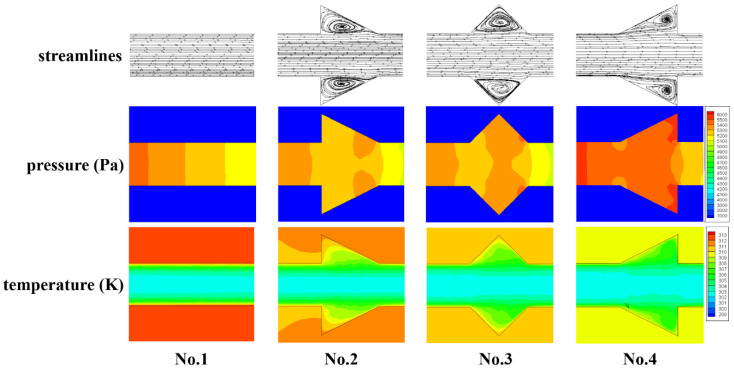
Local streamlines, pressure and temperature distribution of Group 1 and straight microchannel (No. 1).

**Figure 10:**
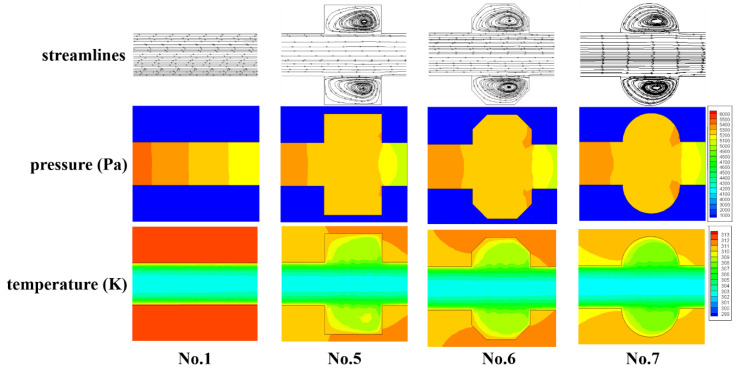
Local streamlines, pressure and temperature distribution of Group 2 and straight microchannel (No. 1).

It is necessary to understand the flow structure and thermal behavior in microchannels before discussing the heat transfer performance. Figure 9 and Figure 10 show the streamlines, pressure distribution and temperature distribution of local heat sink for Group 1 and 2, respectively. The simulation results of the straight microchannel (No. 1) are displayed as a comparison at the same time. It should be mentioned that the flow direction is from left to right; the Reynolds number is 400 and the heat flux is 100,000 $W/m^{2}$. 

As Figure 9 and Figure 10 show, owing to frictional losses, the pressure continuously decreases along the flow direction (*x*-direction) in all microchannels and drops regularly in a smooth microchannel (No. 1). As for the heat sink with cavities, the lowest pressure occurs at the junction of the cavity due to the sudden expansion, while the pressure peak appears at the contraction surface of the cavity. Hence, the reverse pressure gradient occurs inside of the cavities, inducing the vortices as the streamline figure illustrates. Additionally, owing to the reverse pressure gradient, the secondary flows skim over the corner of the cavities to eliminate the slow velocity region causing by sharp corners. From the temperature distribution, one can observe that the temperature of the silicon basement for No. 1 is much higher than that of the other microchannels, indicating that the existence of the cavities can give rise to significant heat transfer enhancement. In general, the temperature difference between silicon basement and the channels could represent the mixture level of the cold fluid near the channel center and the hot water near the sidewalls. As the figures show, the temperature discrepancy is much smaller in No. 4 and No. 7 compared with other microchannels, indicating the effective mixing of working fluid in them. Therefore, it can be concluded that the smaller expansion angle and streamlined edge of the cavities can significantly improve the heat transfer rate compared with other design. This conclusion can be contributed to by the facts discussed above, namely that the smaller expansion angle of the cavities delays the flow separation and avoids the stagnation zone caused by sudden expansion, and the streamlined edge avoids the thick boundary layers caused by sharp corners, increases the vortex area inside the cavities and enhances heat transfer eventually.

#### 3.2.3. Thermal Enhancement Factor

In general, the enhancement of heat transfer is concerned with the increase in fiction factor as a penalty. From the above discussion in the present work, the cavities with smaller expansion angle (No. 4) and streamlined edge (No. 7) can improve the thermal performance of microchannels the most. However, the fiction factors also improved at the same time. Hence, the thermal enhancement factor (*ƞ*) is adopted as the evaluation standard.

**Figure 11:**
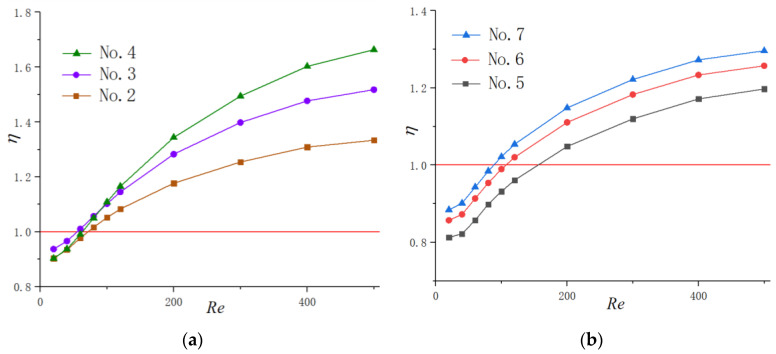
Variation of *ƞ* with Re. (**a**) Group 1, (**b**) Group 2.

Figure 11a,b exhibits the function of the Reynolds number on *ƞ* for Group 1 and 2, respectively. The enhancement factor goes up steadily with the increase in Reynolds number for all microchannels due to the reason discussed above. The discrepancy among different cavity-shaped heat sinks increases with the augment of the Reynolds number. In addition, *ƞ* increases with the diminishing of the expansion angle and catches up to the highest value in the semicircle-shaped cavity, though its heat transfer area is smaller than other heat sinks. This indicates that the negative effect of an increase in fiction factor for No. 4 and No. 7 is overcome by the more dominant positive effect of heat transfer augment. 

#### 3.2.4. New Cavity Shape Design

**Figure 12:**
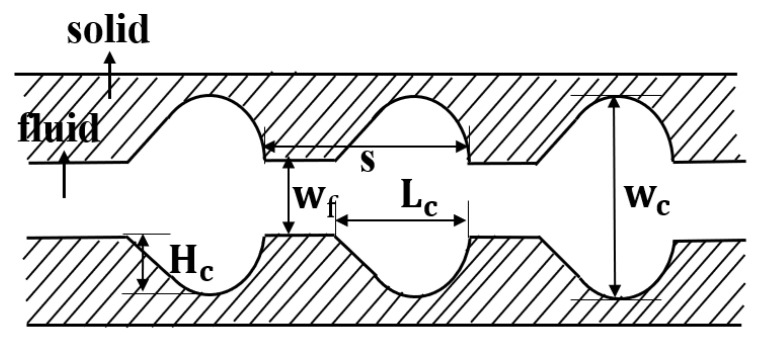
New shape of cavities proposed in this paper.

**Figure 13:**
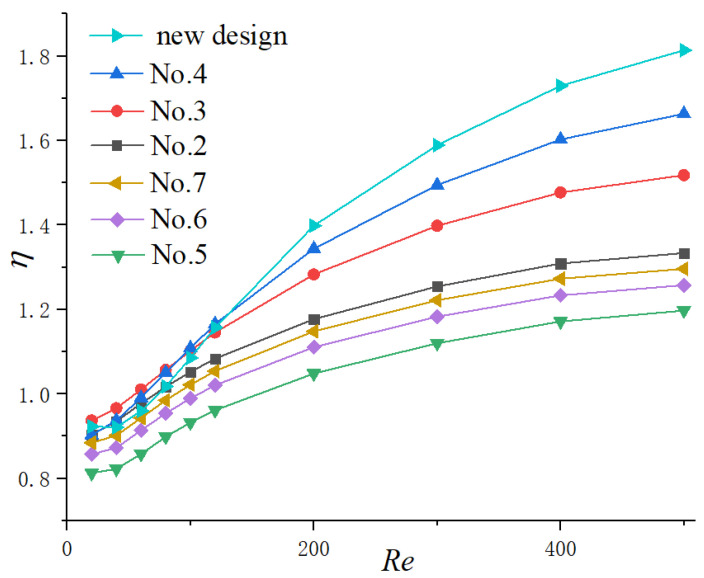
Variation of *ƞ* with Re.

Through the above results and analysis, we have concluded that the existence of cavities could significantly enhance thermal performance in microchannels by interrupting and redeveloping boundary layers as well as spurt and throttling effects. Additionally, the cavity with a relatively smaller expansion angle and streamlined edge can improve the thermal performance to the utmost extent. Hence, a new shape of cavity is proposed in the present work adopting the above conclusions as Figure 12 shows. The expansion angle of the cavity is relatively small, and the arc edge is adopted to avoid sudden expansion and sharp corners, which may cause a thicker thermal boundary layer. Additionally, the straight lines of the cavities are tangent to the arcs to avoid the friction loss caused by tip corners. The parameters keep same with the cavities investigated before, as $W_{c}=0.7\;\mathrm{mm}$, $W_{f}=0.3\;\mathrm{mm}$, $H_{c}=0.2\;\mathrm{mm}$ and s=1.2 mm. Compared with other microchannels in the present work, this shape of cavity is expected to get the highest thermal performance theoretically. Simulations were carried out to verify the assumption and the results are shown in Figure 13. It can be observed that when Re≥120, the thermal enhancement factor of the new proposed cavity is higher than other microchannels at a certain Reynolds number, indicating that the discussion and analysis above is correct. 

**Figure 14:**
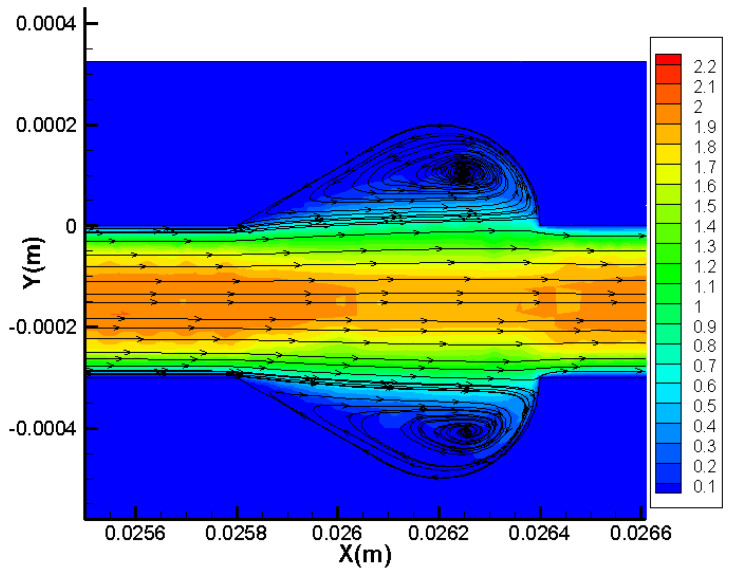
Local velocity distribution and streamlines in new proposed cavity.

Flow fields analysis is performed to explain the thermal performance enhancement of the new proposed cavity shape. The streamlines accompanied with local velocity distribution at the plane of half the fluid depth for *Re* = 400 are shown in Figure 14. As discussed above, the effect of cavities on the enhancement of heat transfer can be contributed to by the following facts. (1) On the one hand, the existence of cavities makes the thermal boundary layer broken and redeveloped continuously. (2) On the other hand, an impinging vortex may generate for higher Reynolds number, which could enhance the fluid mixing degree. Furthermore, a relatively small expansion angle accompanied with the streamlined edge could avoid the thicker boundary layers produced by a sudden expansion and tip. As Figure 14 presents, there is little dead laminar region in cavities, and the vortices filled the full cavities. These flow characteristics result in the excellent thermal performance of this cavity shape. It should be emphasized that this is not the only shape with the best effect of enhancing heat transfer. Instead, all the cavities with small expansion angle and streamline edges could enhance the heat transfer significantly.

From the above analysis and verification, it can be concluded that the cavity with a relatively small expansion angle and streamlined edge can improve the thermal performance of microchannels to the utmost extent.

### 3.3. The Optimized Design for Cavity Parameters

As illustrated in the above research, a new shape of cavity has been proposed that could enhance the thermal performance. In order to figure out the optimum design of the cavity, three design variables that represent aspect ratio, size and spacing, respectively, are simulated, including $\alpha \;\left(=H_{c}/L_{c}\right)$, $\beta \;\left(=W_{c}/W_{f}\right)$, $\gamma \;\left(=s/W_{f}\right)$. As Figure 12 presents, $H_{c}$ and $L_{c}$ is the depth and length for a single cavity, respectively.$\;W_{c}$ is the width of the cross section, $W_{f}$ is the width of the straight channel and s is the cavity spacing.

#### 3.3.1. Optimization Design for the Parameter α

In order to figure out the optimization design for the aspect ratio of the cavity, microchannels are carried out with the dimensionless parameter $\alpha \left(=H_{c}/L_{c}\right)$ ranges from 0.2 to 0.44 with β=2.3 and γ=3. As Figure 12 presents, $H_{c}$ keeps a constant as $H_{c}=0.2\mathrm{mm}$, while $L_{c}$ ranges from 1 mm to 0.45 mm when α ranges from 0.2 to 0.44.

**Figure 15:**
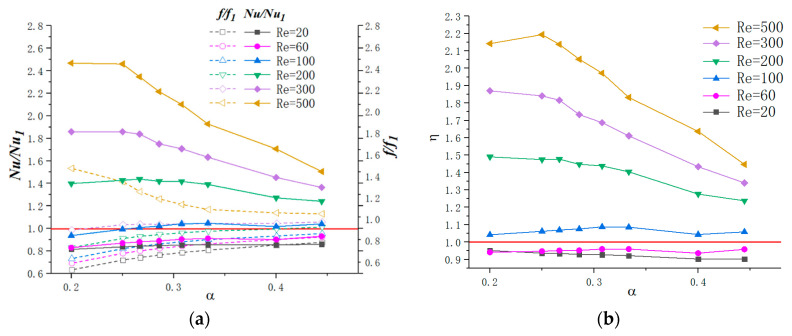
Variation of *f/f*_1_*, Nu/Nu*_1_ and ƞ with *α* for different Reynolds number. (**a**) *f*/*f*_1_, *Nu*/*Nu*_1_, (**b**) *ƞ*.

Figure 15a shows the effect of parameter α on heat transfer and friction factor under different Reynolds numbers. From the simulation results, except for Re=500, the values of $f/f_{1}$ are less or slightly higher than 1, indicating that the new shape of cavity has a positive effect on reducing the friction factor. As for $Nu/Nu_{1}$, the results are different under different flow conditions: (1) When Re≤100, *Nu/Nu*_1_ fluctuate slightly with the augment of α. (2) For Re≥100, the tendency can be divided into two parts: for 0.2≤α≤0.25, *Nu/Nu*_1_ goes up slightly for Re=500, and, for α≥0.25, it declines. The thermal enhancement factor ƞ as a function of α under different Reynolds numbers is shown in Figure 15b. Same as the tendency of *Nu/Nu*_1_*,* ƞ reaches to the highest value for α around 0.2 for Re=200 and Re=300. In terms of Re=500, the optimized value for α is 0.25. In addition, *Ƞ* = 1 is indicated as a red line in figures. Considering the criterion that the thermal performance is enhanced when *Ƞ* ≥1, cavities could be applied to microchannel thermal enhancement for Re≥100 according to the simulation results.

**Figure 16:**
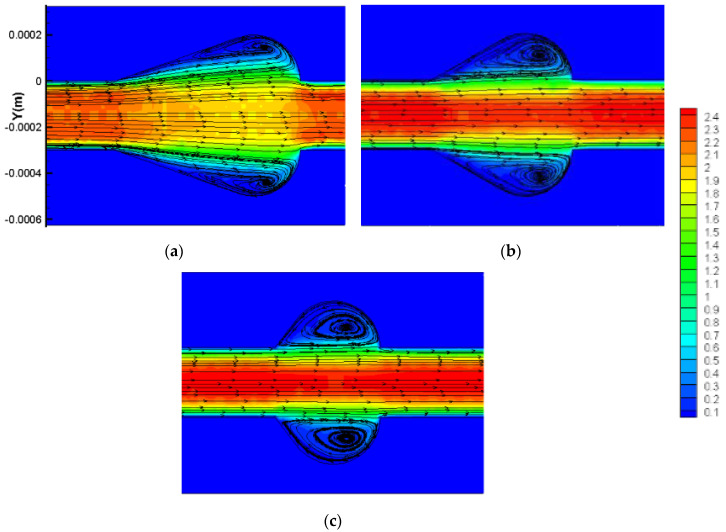
Velocity and streamline distribution in cavities with different α. (**a**) α=0.25, (**b**) α=0.33, and (**c**) α=0.44

It should be mentioned that as $H_{c}$ keeps a constant when increases, which means $L_{c}$ decreases, the expansion angle also enlarges and the cavity area decreases. Hence, the above results could be contributed to the following facts: (1) For Re≤100, as discussed above, the existence of cavities has no positive effect on thermal enhancement performance since the fluid slip over the cavities instead of inducing jetting and spurting effects. (2) For Re≥100, the flow velocity increases and vortices are formed in cavities. Hence, the thermal performance is enhanced as a result. When α increases, as Figure 16 presents, the cavity expansion angle also increases. As discussed above, sudden expansion angle would induce the laminar dead region. Hence, the thermal enhancement factor declines with the augment of α. (3) Since the jetting and spurting effects are more intense for higher Reynolds numbers, the positive effect of cavities on thermal enhancement is more significant under relatively higher Reynold numbers. In addition, the optimal design value of α for Re=500 is larger than that of 100≤Re≤300, which may be contributed to by the fact that a higher flow velocity requires a relatively larger expansion angle to form vortices. 

From the above analysis, for Re≤100, in order to pursue higher thermal performance, the parameter α should as large as possible; for 100≤Re≤300, the optimal criterion is α=0.2; for Re=500, the optimal parameters α=0.25; for a higher Reynolds number, the optimal valves of α are even larger.

#### 3.3.2. Optimization Design for the Parameter β

For a cavity when $W_{f}$ and α are constant, $\beta \;\left(=W_{c}/W_{f}\right)$ determines the size of the cavities as Figure 12 shows. Simulations are carried out to determine the optimized design for dimensionless parameter *β*.

**Figure 17:**
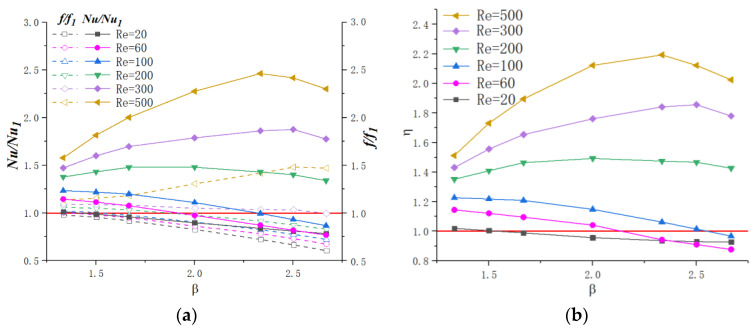
Variation of *f/f*_1_, *Nu/Nu*_1_ and ƞ with β for different Reynolds number. (**a**) *f*/*f*_1_, *Nu*/*Nu*_1_, (**b**) *ƞ*.

Figure 17a presents the effect of parameter *β* on heat transfer and friction factor under different Reynolds numbers. The dimensionless parameter *β* ranges from 1.33 to 2.67 with α=0.25 and γ=3. As the figure shows, for Re≤100, as *β* increases, *f/f*_1_ and *Nu/Nu*_1_ decreases linearly. When 200≤Re≤300, *f/f*_1_ declines slightly with the augment of *β* while for Re=500, *f/f*_1_ increases. For *Nu/Nu*_1_, the tendency could be divided into three parts when Re≥100: 1.33–1.67, 1.67–2.33, 2.33–2.67. As *β* increased, the *Nu/Nu*_1_ rises rapidly for β=1.33−1.67, slowly increase and even drop a small margin for β=1.67−2.33 and declines a large scope for β=2.33−2.67. The above tendencies of *f/f*_1_ and *Nu/Nu*_1_ induce the variation of ƞ with β for different Reynolds number values, as Figure 17b presents. For Re≤100, *Ƞ* declines with the augment of β. In terms of Re≥100, ƞ reaches peak values for β=2.0−2.5. In addition, the values of *Ƞ* are much higher for relatively higher Reynolds numbers, indicating that the positive effect of cavities on thermal enhancement is more significant in higher velocity flow conditions.

**Figure 18:**
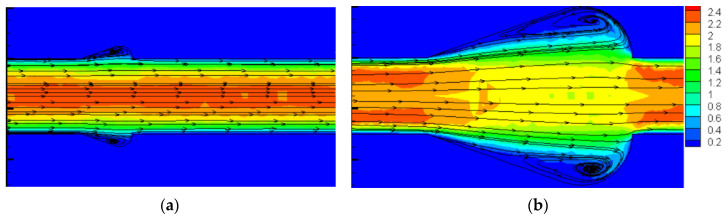
Velocity and streamline distribution in cavities for Re=500  with different β. (**a**) β=1.33 (**b**) β=2.33.

Figure 18 presents the flow characteristics of local cavities for β=1.33 and β=2.33 for *Re* = 500. As display in Figure 18, the above results could be contributed to the following facts: (1) When β=1.33−1.67, the cavities are too small to bring about the strong vortices that can improve the thermal performance. In addition, the boundary layer cannot be interrupted and the cavities buried in boundary layer instead. Owing to these situations, the heat transfer performance may be weakened. When β increases, the size of the vortex becomes larger and the jetting effect turns to be more obvious. Hence, the thermal performance effect improves significantly as a result. (2) For β=1.67−2.33, the larger the Reynolds number is, the more intense jetting and spurting effect attend. Therefore, for relatively high flow velocity conditions, the optimization design for β also increases, since a larger space is needed to form a vortex. (3) When β>2.33, the space of the cavities is much larger than the vortices need. Therefore, a laminar dead region may be formed, which has a negative effect on heat transfer. Hence, the value of ƞ declines remarkably. (4) In terms of the condition that Re≤100, the simulation results are consistent with the above research that adding cavities has no improvement on thermal performance. When β increases, the effect of cavities enhances significantly, resulting in a decrease of ƞ. 

Therefore, for Re≤100, in order to pursue higher thermal performance, β should be as small as possible, meaning that the straight microchannel is optimal. As for Re≥100, a moderate increase of β can enhance the thermal performance in microchannels, and the optimized value is β=2.0−2.5.

#### 3.3.3. Optimization Design for the Parameter γ

**Figure 19:**
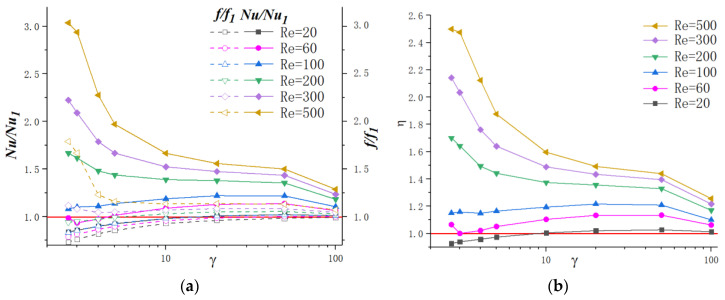
Variation of *f/f*_1_*, Nu/Nu*_1_ and ƞ with γ for different Reynolds number. (**a**) *f*/*f*_1_, *Nu*/*Nu*_1_, (**b**) *ƞ*.

Figure 19a presents the effect of cavity spacing on friction factor and Nusselt number under different Reynolds numbers. In order to figure out the discrepancy between simple straight microchannels, the values of Nusselt number (*Nu*) and friction factor (*f*) are divided by the values of straight microchannels (No. 1). The parameter values are given as α=0.25 and β=2.0, with γ ranging from 2.67 to 100, and the values of γ and cavities numbers are shown in Table 2. The values of γ and corresponding numbers of cavities are shown in Table 2.

As discussed above, adding cavities on microchannels can hardly improve the thermal performance for relatively lower Reynolds numbers. From the tendency of *Nu/Nu*_1_ and *f/f*_1_ in Figure 19a, it can be observed that, for relatively lower Reynolds number (*Re* = 20, 60), a moderate value of γ could make the heat transfer ability exceeds that of a straight microchannel. However, when γ goes up to γ = 100, the heat transfer performance goes back down close to the straight microchannel. For other Reynolds numbers, *Nu/Nu*_1_ increases significantly with an acceptable pressure loss when γ decreases. For instance, when γ=4, the Nusselt number can reach 2.27 times higher than that of simple straight microchannels for *Re* = 500. As for γ=100, this value turns to be only 1.29 under the same Reynolds number. This phenomenon can be explained that when γ decreases, the quantity of vortex production in cavities increases, which may enhance the heat transfer. It can also be noted that the thermal performance improvement degree is more remarkable for a higher Reynolds number. For example, when γ=5, the Nusselt number is 1.87 times for *Re* = 500, and only 1.44 for *Re* = 200. 

Based on the above analysis, the effect of γ can be concluded as follows: (1) For lower Reynolds (within 100 in the present work), the existence of cavities can hardly improve heat transfer. However, it could reduce the friction factor on some level. With the increase in γ, the thermal performance and flow resistance approach to that of straight microchannels is gradual. (2) For relative higher laminar Reynolds numbers, cavities can enhance heat transfer and the enhancement degree turns to become more significant as Reynolds number increases. This can be contributed to the fact discussed above that higher flow velocity induces more intense vortex and thinner boundary layers, which may enhance the thermal performance.

From the above discussion, when Re≤100, the development of thermal performance increases with the augment of γ. For higher Reynolds number, decreasing γ can enhance heat transfer significantly, and 2≤γ≤3 is the optimization criterion for the spacing of cavities.

## 4. Conclusions

In this study, microchannel exchangers with different cavities have been investigated to determine the influence mechanism of cavity shape. Based on the conclusions, a new cavity shape is proposed. Further simulations are performed additionally to investigate the optimized geometric parameters of the cavity. With respect to the simple straight microchannel, the following conclusions can be drawn from the present study:

The influence of cavities to the flow resistance and thermal performance varies based on the Reynolds number of the flow. For relatively lower Reynolds numbers (*Re* < 100 in this study), there are no vortices formed in cavities due to the low flow velocity, and adding cavities on microchannel sidewalls has no positive effect on thermal enhancement; hence, straight microchannels are optimal for microchannel thermal design. However, when the Reynolds number increases, heat transfer is enhanced since the vortices are formed inside the cavities, which can interrupt the thermal boundary layer and make the fluid mix more intense. As for the critical Reynolds number that happens during the transformation of a negative effect into a positive effect on thermal performance, different shapes of cavities keep different values.

Thermal performance can be enhanced by controlling cavity shapes. On the one hand, the smaller expansion angle of the cavities could delay the flow separation and avoid the low velocity zone caused by sudden expansion. On the other hand, the streamlined edge avoids the thick boundary layers caused by sharp corners and increases the vortex area inside the cavities at the same time. Therefore, a relatively small expansion angle accompanied with a streamlined edge are required for cavities’ shape design to improve the thermal performance.

A new shape of microchannel cavity with high heat transfer and low flow resistance was designed, which could be applied to microchannel exchangers. The optimal geometric parameters of cavity under different flow conditions have been obtained in principle for microchannel exchangers design. The parameter α determines the aspect ratio of cavities. α should be as large as possible for Re≤100 in this present work. α=0.2 for 100≤Re≤300. For Re=500, the optimal parameters are α=0.25, and, for higher Reynolds numbers, the optimal valves of α are even larger. The parameter *β* determines the size of the cavities. β should be as small as possible for Re≤100 in order to pursue high thermal performance and 2.0≤β≤2.5 for 100≤Re≤500. The parameter γ determines the quantity and spacing of cavities and should be as high as possible for Re≤100 while 2≤γ≤3 for 100≤Re≤500.

## Figures and Tables

**Table 1:** Grid independence test.

No.	Cell Numbers $\times 10^{6}$	*Nu*	e%	Pressure Drop/Pa	e%
1	0.96	4.31	3.58	6358.83	4.01
2	1.86	4.38	2.01	6441.76	2.76
3	2.97	4.47	0	6620.92	0.55
4	4.78	4.47	-	6624.58	-

**Table 2:** Values of γ and corresponding numbers of cavities.

γ	2.67	3	4	5	10	20	50	100
Number of Cavities	48	43	32	26	13	7	3	1

## Nomenclature

L	length of the microchannel, m
$W_{s}$	width of the silicon basement, m
$H_{s}$	height of the silicon basement, m
$W_{f}$	width of the microchannel, m
$H_{f}$	height of the microchannel, m
$u_{in}$	velocity component at the microchannel inlet, m/s
$T_{f}$	temperature of the fluid, K
$T_{in}$	temperature at the microchannel inlet, K
$p_{f}$	static pressure of the fluid, Pa
$p_{out}$	static pressure at the microchannel outlet. Pa
u	velocity component in the x direction, m/s
v	velocity component in the y direction, m/s
w	velocity component in the z direction, m/s
$T_{s}$	temperature of the silicon basement, K
$q_{w}$	heat flux per area, W/$m^{2}$
s	cavity spacing, m
$L_{c}$	longitudinal length of a single cavity, m
$H_{c}$	height of a single cavity, m
$W_{c}$	total width of the microchannel plus cavities, m
x, y, z	three coordinates shown in Figure 1, m
ρ	density, kg/m^3^
$\mu_{f}$	dynamic viscosity, Pa s
$C_{pf}$	specific heat of fluid, J Kg^−1^ K^−1^
$k_{f}$	thermal conductivity of fluid, W m^−1^ K^−1^
$k_{s\;}$	thermal conductivity of silicon, W m^−1^ K^−1^
Re	Reynolds number
*f*	friction factor
*Nu*	Nusselt number
*Ƞ*	thermal enhancement factor
$u_{m}$	average velocity, m/s
$D_{h}$	hydraulic diameter, m
∆p	pressure drop, Pa
*h*	heat transfer coefficient, W m^−2^ K^−1^
A	area of contact surface between the fluid zone and solid zone
n	local coordinate normal to the wall
*AR*	aspect ratio
$f_{FD}$	friction factor of fully developed flow
